# Outcomes measured by mortality rates, quality of life and degree of autonomy in the first year in stroke units in Spain

**DOI:** 10.1186/s12955-015-0230-8

**Published:** 2015-03-17

**Authors:** Javier Mar, Jaime Masjuan, Juan Oliva-Moreno, Nuria Gonzalez-Rojas, Virginia Becerra, Miguel Ángel Casado, Covadonga Torres, María Yebenes, Manuel Quintana, Jose Alvarez-Sabín, on behalf of CONOCES Investigators Group

**Affiliations:** Clinical Management Service, Alto Deba Hospital, Mondragon, Spain; Stroke Unit, Neurology Department, Hospital Universitario Ramón y Cajal. Universidad de Alcalá, Madrid, Spain; Department of Economic Analysis, Universidad de Castilla La Mancha and REDISSEC, Toledo, Spain; Health Economics and Outcomes Research, Boehringer Ingelheim España, Barcelona, Spain; Pharmacoeconomics & Outcomes Research Iberia, Madrid, Spain; Neurovascular Unit, Department of Neurology, Universitat Autònoma de Barcelona, Hospital Vall d’Hebron, Barcelona, Spain; Unidad de Gestión Sanitaria, Hospital ‘Alto Deba’, Avenida Navarra 16, 20500 Mondragón, Spain

**Keywords:** Stroke, Outcomes, Disability, Quality of life, Stroke units, Thrombolysis

## Abstract

**Introduction:**

The primary objective of this sub analysis of the CONOCES study was to analyse outcomes in terms of mortality rates, quality of life and degree of autonomy over the first year in patients admitted to stroke units in Spain. The secondary objective was to identify the factors determining good prognosis.

**Methods:**

We studied a sample of patients who had suffered a confirmed stroke and been admitted to a Stroke Unit in the Spanish healthcare system. Socio-demographic and clinical variables and variables related to the level of severity (NIHSS), the level of autonomy (Barthel, modified Rankin) and quality of life (EQ-5D) were recorded at the time of admission and then three months and one year after the event. Factors determining prognosis were analysed using logistic regression and ROC curves.

**Results:**

A total of 321 patients were recruited, 33% of whom received thrombolytic treatment, which was associated with better results on the Barthel and the modified Rankin scales and in terms of the risk of death. Mean quality of life measured through EQ-5D improved from 0.57 at discharge to 0.65 one year later. Full autonomy level measured by Barthel index increased from 30.1% at discharge to 52.8% at one year and by the modified Rankin scale from 51% to 71%. The rates for in-hospital and 1-year mortality were 5.9% and 17.4% respectively. Low NIHSS scores were associated with a good prognosis with all the outcome variables. The three instruments applied (NIHSS, Barthel and modified Rankin scales) on admission showed good discriminative ability for patient prognosis in the ROC curves.

**Conclusions:**

There has been a change in the prognosis for stroke in Spain in recent years as the quality of life at 1 year observed in our study is clearly higher than that obtained in other Spanish studies conducted previously. Moreover, survival and functional outcome have also improved following the introduction of a new model of care. These results clearly promote extension of the model based on stroke units and reinforced rehabilitation to the majority of the more than 100,000 strokes that occur annually in Spain.

## Introduction

In the 20th century, specific treatment for stroke was mainly directed at primary prevention, with a particular focus on controlling risk factors like hypertension [1-3]. While patients were in hospital, the clinician’s role was limited to maintaining vital functions and preventing complications [4]. The advent of thrombolysis and its successful use in acute myocardial infarction opened the way for an active approach to patient care, which led to the creation of stroke units [5-7]. At the same time the Stroke National Plan reinforced the key role of rehabilitation in the final outcome [8,9]. With this change in the model of care, it was recognised that the care received during hospitalisation and in the first year is a critical element in the prognosis of patients with stroke, both in terms of survival and functional status, and thus a determining factor in the resulting economic and social burden [7]. The new pro-active approach enabled the previous fatalistic attitude to be overcome and led to improved outcomes through the incorporation of evidence-based therapies such as reperfusion and stroke units into the standard treatment [1,7-10]. Thus, the healthcare system placed emphasis on the characteristics of the different care levels with promotion of reference stroke hospitals, set up less restrictive stroke code activation criteria that included new therapeutic options, established new standard measures for endovascular treatment, reinforced the rehabilitation process and developed tele-medicine stroke networks [8,9].

Current demographic trends towards aging mean that measurement of the burden of disease has had to give greater priority to diseases which, in addition to causing mortality, also generate disability [11,12]. As a result, health-related quality of life (HRQOL) has become a key element when measuring outcomes of medical interventions [13,14]. However, most of the studies carried out in Spain on the impact of stroke on HRQOL are dated from several years ago [15-17]. It is therefore necessary to measure the impact that the widespread introduction of stroke units has had on patients’ HRQOL and level of autonomy, in order to assess the improvements made to secondary and tertiary stroke prevention [18].

The primary objective of this part of the CONOCES study [19] was to analyse outcomes in terms of mortality rates, health-related quality of life (HRQOL) and degree of autonomy in patients admitted to stroke units in Spain over the first year after admission. The secondary objective was to identify the factors determining good outcome.

## Methods

### Type of study and design

CONOCES was a prospective, observational, epidemiological, naturalistic, multicentre study of costs and outcomes of the disease in the Spanish healthcare setting in a sample of patients who had suffered a confirmed stroke [19]. The inclusion criteria were: being over 18 years of age; confirmed clinical diagnosis of first ischaemic or haemorrhagic stroke within 24 hours of onset; admission to a stroke unit; voluntary participation in the study; and signed informed consent by the patient and/or their primary caregiver. During the recruitment phase patients who met the inclusion criteria were enrolled consecutively in the study in all the participant centres. The study variables were collected by the medical team through personal interviews with the patient and the primary caregiver during the hospital stay in the stroke units and at the follow-up visits at 3 and 12 months. In the first data collection all the autonomy and quality of life scales were recorded at hospital discharge. Only the mRS was also recorded at admission. The protocol was approved by the Ethics Committee of Hospital Clínic i Provincial de Barcelona.

The variables were socio-demographic (age, gender and residence) and clinical (activation of stroke protocol, time to neurological care, aetiology, recurrence, presence of atrial fibrillation (AF), thrombolytic therapy and death during the follow-up period) in nature. HRQOL and degree of autonomy, as well as their determining factors, were also measured by determining neurological status on admission using the National Institutes of Health Stroke Scale (NIHSS) [20] and by assessing the patients’ disability by measuring functional status with the Barthel Index (BI) [21] and degree of functional dependence with the modified Rankin Scale (mRS) at discharge, three months and one year [22]. BI and mRS are the most commonly used measurement scales to assess the level of autonomy of stroke patients. mRS has become the most widely used clinical outcome measure for stroke in clinical trials. The scale ranks from 0 to 6, from perfect health with no symptoms to death [22]. Although the mRS classifies deaths in category 6, we decided to exclude them from the functional status analyses. BI is an ordinal scale used to measure performance in daily living activities. Each performance item is rated on this scale with a given number of points assigned to each level or ranking. It uses ten variables to describe mobility, feeding, toilet use, dressing, bathing, faecal and urinary incontinence, help needed with grooming and other activities. A higher number is associated with a greater likelihood of being able to live at home with a degree of independence following discharge from hospital. Each item can score 0, 5 or 10 points, so that BI score range from 0 to 100, corresponding to five levels of dependence: independent (100 points), low dependence (91–99 points), moderate dependence (61–90 points), severe dependence (21–60 points), total dependence (0–20) [21,23]. NIHSS has become the most widely-used scale to assess initial neurological status, in the follow-up of patients on treatment and also for routine clinical monitoring in stroke units [24,25]. It is composed of 11 items, each of which scores a specific ability between 0 and 4. For each item, a score of 0 typically indicates normal function in that specific ability, while a higher score is indicative of some level of impairment. The individual scores from each item are summed in order to calculate a patient’s total NIHSS score. The maximum possible score is 42, with the minimum score being 0 [24,25].

HRQOL was measured using the generic questionnaire EuroQuol (EQ-5D) at the same three time points [26]. The EQ-5D questionnaire was completed by the patients at the scheduled follow-up visits for the study. EQ-5D is a standardised instrument to measure self-reported health status with respect to five dimensions (mobility, self-care, usual activities, pain/discomfort, anxiety/depression) and three levels of severity (no problems, some or moderate problems, and extreme problems). It is characterised by the fact that it provides a single index value for each health state which can have a value of 1 or less, where 1 is equivalent to full health and 0 is death. These were normalised using conversion tables obtained in the general Spanish population [27]. When the patient was unable to communicate, the interviewer conducted the test to a close relative or caregiver [28].

### Statistical analysis

Statistical differences in the comparison of contingency tables were analysed using the chi-square test, taking a value of α equal to or less than 0.05. Given that the variables are not normally distributed, the Wilcoxon test was used to measure the statistical significance of differences between means, as with ordinal data. All statistical analyses were carried out using SPSS software (version 21; SPSS Inc., Chicago, IL).

To analyse the magnitude of the observed change in the quality of life, the value of the final EQ-5D was compared with the original and with a sample of general population over 65 using the effect size [29]. The EQ-5D sample used to describe general population characteristics proceeded from a random sample of general population from the Canary Islands [30] which mean score was 0.73 and standard deviation 0.32. Cohen defined as non-significant an effect size of less than 0.2, an effect size between 0.2 and 0.5 as small, between 0.5 and 0.8 as moderate, and greater than 0.8 as large [29].

To analyse the association of clinical variables on admission with functional capacity and HRQOL after a year, statistical logistic regression analyses were performed in which the dependent variables were mortality, the value 1 in EQ-5D, modified Rankin Scale level 0–2, Barthel below 61 (total or severe dependence) and Barthel 100. The independent variables were age, gender, arrival time to neurological care, aetiology (ischaemic or haemorrhagic), AF, NIHSS, hospital arrival time and thrombolytic therapy (yes/no). The likelihood of receiving thrombolytic therapy was further investigated in a logistic regression model in which the explanatory variables were age, gender, NIHSS and the time of arrival.

Furthermore, we used an approach based on the analysis of ROC (Receiver Operating Characteristic) curves to measure sensitivity to change. This allowed us to assess the ability of the instruments used on admission, such as the NIHSS, the mRS and the BI, to detect patients with good prognosis, using the same methodology that is used to determine whether or not a diagnostic test identifies the condition of interest [31]. The ROC curve is obtained by plotting sensitivity for each breakpoint on the y-axis and its corresponding 1-specificity on the x-axis. If the instrument is sensitive, it has an ROC curve where sensitivity peaks have associated minimum values of 1-specificity. Conversely, if the instrument is insensitive, it has an ROC curve near to the reference line (line dividing the axis into two equal parts, which indicates the minimum level of discrimination). To characterise the instrument as discriminant, areas greater than 70% are required. The statistical method also calculated the statistical significance of the curve.

## Results

A total of 321 patients were enrolled in the study between November 2010 and May 2011. Eight patients were lost to follow-up between discharge and the 3-month visit, and a further 7 by 12 months. The mean age was 72.1 years (SD 13.2) and 176 patients (54.8%) were male. The main socio-demographic and clinical characteristics of the sample are shown in Table 1. We should highlight that 33% of patients received thrombolytic therapy. The rates for in-hospital and 1-year mortality were 5.9% and 17.4% respectively.

**Table 1:** **Socio-demographic and clinical characteristics of the patients**

**Age in years**	**Mean (SD)**	**72.1 (13.2)**
**Gender**	Value	(%)
Male	176	54.8
Female	145	45.2
**Living Environment**	Value	%
Home	316	98.4
Residential Care for the Elderly	3	0.9
Other	2	0.6
**Stroke Protocol Activated**	Value	%
No	147	45.8
Yes	167	52
**Time to neurological care**	Frequency	Percentage
0-1.5 hours	71	22.7
1.5-3 hours	94	30
3-4.5 hours	34	10.9
>4.5 hours	114	36.4
**Type of stroke**	Value	%
Cerebral Infarction	291	90.7
Haemorrhage	30	9.3
**Stroke recurrence at 1 year**	Value	%
Yes	25	7.8
No	296	92.2
**Atrial Fibrillation**	Value	%
Yes	161	50.2
No	160	49.8
**Thrombolysis total**	Value	%
Yes	106	33.0
No	215	67.0
**Thrombolysis NIHSS** 6-42	Value	%
Yes	92	50.8
No	89	49.2
**Thrombolysis NIHSS** 0-5	Value	%
Yes	14	10.0
No	126	90.0
**Death while in hospital**	Value	%
Yes	19	5.9
No	302	94.1
**Death during the year of study**	Value	%
Yes	56	17.4
No	265	82.6
**NIHSS on admission**	Value	%
Minor strokes (1–3)	86	26.8
Moderate strokes (4–10)	114	35.5
Severe strokes (>10)	121	37.7

NIHSS: National Institute of Health Stroke Scale.

**Figure 1:**
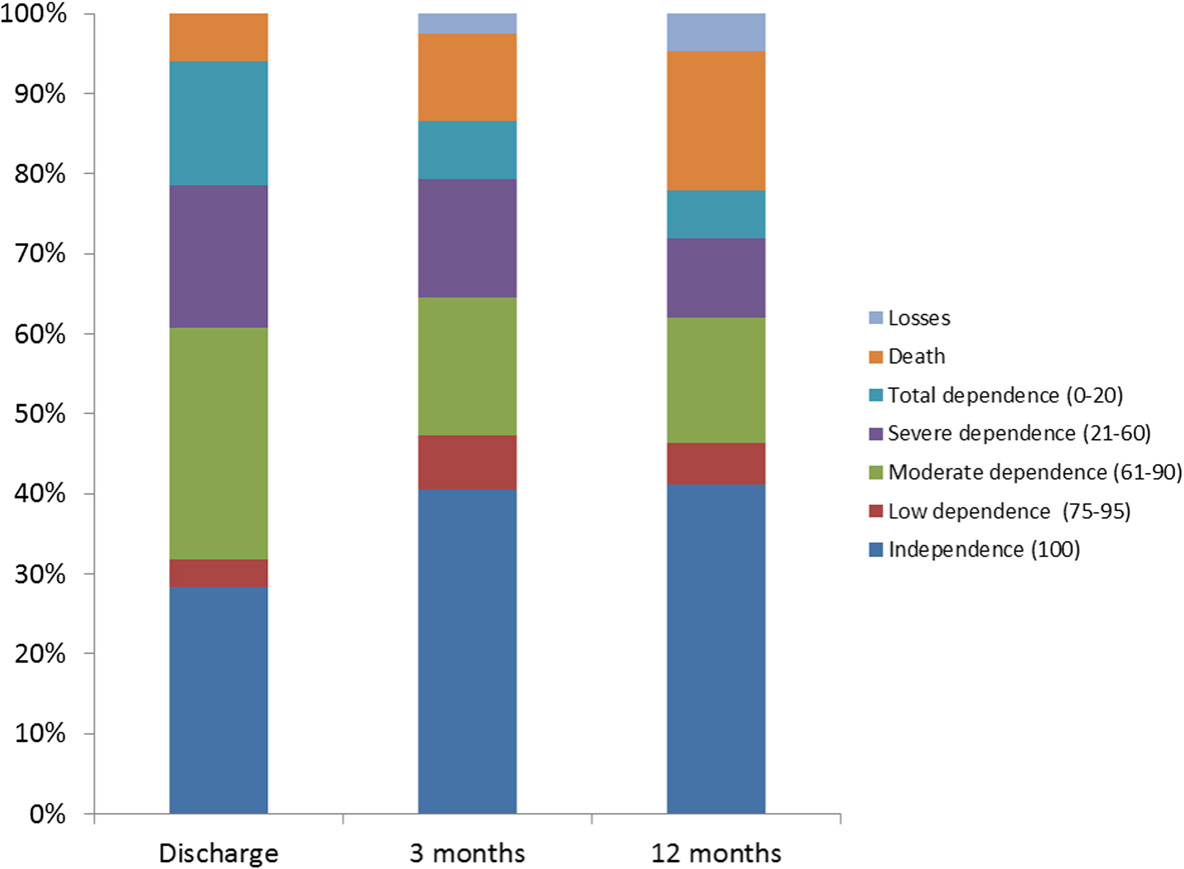
**Frequency histogram of the level of dependence from admission up to 12 months.**

The level of autonomy and quality of life from admission to one year later improved on all scales (Table 2). The percentage of category 3 (no problems) in the dimensions of the EQ-5D increased across the board. Figure 1 shows how functional level evolved according to the BI from admission to 12 months. The effect size for EQ-5D obtained from hospital discharge to 12 months was small (0.25). When we compared the EQ-5D values of general population with the mean value at 12 months the same effect size was attained. However the Visual Analogic Scale scores did not show any statistically significant differences between the admission and after one year.

**Table 2:** **Changes in level of autonomy and quality of life scales from hospital admission up to 1 year later**

	**Previous**	**Discharge**	**3 months**	**12 months**
**Modified rankin scale**	**N (%)**	**N (%)**	**N (%)**	**N (%)**
From 0 to 2	299 (93.7%)	165 (54.6%)	186 (67.1%)	182 (71.1%)
Above 2	20 (6.3%)	137 (45.4%)	91 (32.9%)	68 (28.9%)
**Barthel**		N (%)	N (%)	N (%)
Independent (100)		91 (28.3%)	130 (40.5%)	132 (41.1%)
Low dependence (91–99)		11 (3.4%)	22 (6.9%)	17 (5.3%)
Moderate dependence (61–90)		93 (29.0%)	55 (17.1%)	50 (15.6%)
Severe dependence (21–60)		57 (17.8)	48 (15.0%)	32 (10.0%)
Total dependence (0–20)		50 (15.6%)	23 (7.2%)	19 (5.9%)
**Cumulative deaths**		19 (5.9%)	35 (10.9%)	56 (17.4%)
**EQ-5D**		**Discharge**	**3 months**	**12 months**
Utilities mean (SD)		0.57 (0.32)	0.62 (0.30)	0.65 (0.28)
Visual Analogic Scale mean (SD)		45.81 (28.62)	44.15 (31.56)	45.74 (33.36)
Number of responses		274	261	234
**EQ-5D dimensions**		**N (%)**	**N (%)**	**N (%)**
Mobility	No problems	122 (44.2%)	137 (52.5%)	123 (52.1%)
	Some problems	100 (36.2%)	98 (37.5%)	100 (42.4%)
	Confined to bed	54 (19.6%)	26 (10.0%)	13 (5.5%)
Personal care	No problems	137 (49.8%)	156 (59.8%)	155 (67.7%)
	Some problems	75 (27.3%)	36 (13.8%)	52 (22.0%)
	Unable	63 (22.9%)	69 (26.4%)	29 (12.3%)
Activity	No problems	83 (30.1%)	116 (44.4%)	116 (49.2%)
	Some problems	122 (44.2%)	44 (16.9%)	84 (35.6%)
	Unable	71 (25.7%)	101 (38.7%)	36 (15.3%)
Pain	No pain	171 (62.4%)	159 (60.9%)	140 (59.3%)
	Moderate pain	94 (34.3%)	88 (33.7%)	86 (36.4%)
	Extreme pain	9 (3.3%)	14 (5.4%)	10 (4.2%)
Anxiety	Not anxious	153 (55.8%)	131 (50.2%)	134 (56.8%)
	Moderately anxious	103 (37.6%)	102 (39.1%)	89 (37.7%)
	Extremely anxious	18 (6.6%)	28 (10.7%)	13 (5.5%)
**Cumulative losses**		0	8	15

In the univariate analysis (Table 3), age, NIHSS on admission and the presence of AF appear as variables statistically associated with level of autonomy (Barthel and modified Rankin scores at 1 year). In contrast, aetiology, thrombolysis and time of arrival are not statistically significant. Male gender is statistically associated with a Barthel scale score of 100 but not with a modified Rankin between 0 and 2.

**Table 3:** **Distribution of variables for level of autonomy at 12 months according to clinical characteristics**

	**Barthel**			**Modified rankin scale**		
	**100**	**0-95**	**p**	**0-2**	**3-5**	**p**
**Gender (Male)**	87 (65.9%)	52 (44.1%)	0.001	107 (58.8%)	32 (47.1%)	NS
**Age < 75 years**	86 (65.2%)	58 (49.2%)	0.015	26 (38.2%)	118 (64.8%)	0.015
**NIHSS on admission > 5**	50 (37.9%)	77 (65.3%)	<0.001	74 (40.7%)	53 (77.9%)	<0.001
**Infarction vs. Haemorrhage**	120 (90.9%)	106 (89.8%)	NS	165 (90.7%)	61 (89.7%)	NS
**Thrombolysis**	42 (31.8%)	40 (33.9%)	NS	57 (31.3%)	25 (36.8%)	NS
**Arrival time < 3 h**	66 (51.2%)	59 (51.8%)	NS	90 (50.6%)	35 (53.8%)	NS
**Atrial Fibrillation**	52 (39.4%)	64 (54.2%)	0.022	76 (41.8%)	40 (58.8%)	0.022

NIHSS: National Institute of Health Stroke Scale.

The results of logistic regression analysis are presented in Table 4 and show that low NIHSS is associated with good prognosis for all outcome variables. Being under the age of 75 was only associated with a better outcome for mortality and low modified Rankin score. Males had better quality of life and increased autonomy in terms of Barthel 100. Although in the univariate analysis the prognosis in patients treated with thrombolysis was found to be no different (Table 3), in the logistic regression, this treatment appears to be associated with better outcome for the Barthel and modified Rankin scores and the risk of death (Table 4). The reason for this difference is the distribution of patients according to NIHSS and thrombolysis, as half of the patients who were in the 6–42 group of the NIHSS received thrombolytic therapy while only 10% of those in the 0–5 group did so (Table 1).

**Table 4:** **Results from logistic regressions relating the characteristics of stroke patients and the 1-year outcome**

	**Barthel 100**		**mRS 0-2**		**EQ-5D 1**		**Barthel <60**		**Death**	
	**p**	**OR**	**p**	**OR**	**p**	**OR**	**p**	**OR**	**p**	**OR**
Haemorrhage	NS		NS		0.046	0.261	NS		NS	
Atrial Fibrillation	NS		NS		NS		NS		NS	
Age over 75	NS		0.001	0.335	NS		NS		0.000	6.196
NIHSS (0–5)	0.000	3.882	0.000	6.985	0.010	2.769	0.000	0.234	0.000	0.201
Male	0.007	2.137	NS		0.005	2.723	NS		NS	
Thrombolysis	0.047	2.173	0.036	2.540	NS		NS		0.006	0.307
Arrival time	NS		NS		NS		NS		NS	

mRS: modified Rankin Scale. OR: Odds ratio. NIHSS: National Institute of Health Stroke Scale.

**Figure 2:**
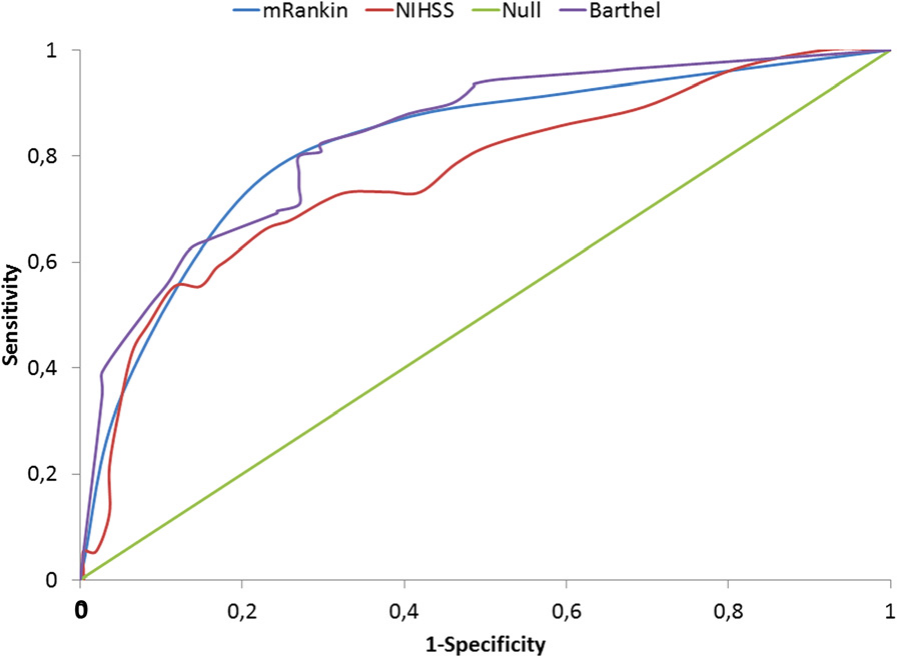
**ROC curves measuring the discriminatory capacity of the modified Rankin, NIHSS and Barthel instruments applied on admission with the prognosis of 1-year mortality.** NIHSS: National Institute of Health Stroke Scale.

The explanatory logistic regression of the probability of thrombolysis showed statistically significant values for a mild NIHSS (OR 8.3) and time of arrival less than 3 hours (OR 9.5). No statistically significant associations were found with age or gender. The ROC curves reached areas under the curve above 70% and statistical significance levels below 0.05 for the three instruments used on admission (NIHSS, Barthel and modified Rankin scales) (Table 5 and Figure 2).

**Table 5:** **Area under the curve and significance values for the ROC curves measuring the discriminatory capacity of the instruments applied on admission with outcome at 1 year**

**1-year outcome**	**Barthel =100**		**Rankin 0-2**		**EQ-5D**		**Barthel <60**		**Death 1 year**	
Instrument on admission	AUC	p	AUC	p	AUC	p	AUC	p	AUC	p
NIHSS	0.677	0.000	0.717	0.000	0.670	0.000	0.671	0.000	0.765	0.000
Barthel			0.856	0.000	0.718	0.000			0.835	0.000
modified Rankin	0.779	0.000			0.737	0.000	0.598	0.028	0.811	0.039

AUC: area under the curve. p: statistical significance value.

## Discussion

The main conclusion from the results of this study is that the prognosis of stroke in Spain has changed in recent years. Both survival and functional outcome have improved as a result of the introduction of a new model of care [18,32]. This new model, based on stroke units, thrombolysis, physical therapy and secondary prevention, has been promoted by the stroke strategy of the Spanish National Health Service in coordination with regional health authorities and healthcare professionals [8,9].

The in-hospital mortality rate (5.9%) is well below the 10% reported in 2006 in the U.S [32]. Compared to the 30% 1-year mortality rate described in the literature, this figure has dropped to 18.3% [18,32]. Also striking is the sequence of disability-scale measurements that shows a functional recovery process in which good levels of autonomy (0–2 on the modified Rankin scale) increase from 51% at discharge from hospital to 71% in the survivors at 1 year. These good results after discharge show that the development of post-stroke rehabilitation and the inclusion of physical therapy in the Spanish Stroke Plan improved the prognosis [8,9].

Thrombolytic therapy is a good prognostic factor, as treated patients were found to have statistically significant OR above 2 in the Barthel and modified Rankin scales in survival. Among the factors that have changed from 10–15 years ago is the time to arrival at hospital, with the median dropping from 6 hours to 3 hours. Along with stroke unit care, this explains why a third of patients received thrombolysis. Multivariate analysis made it possible to see its association with better prognosis despite the NIHSS variable acting as a confounding factor. A low NIHSS score generally corresponds to patients with neurological deficit that is only slightly disabling, and is one of the exclusion criteria in summaries of product characteristics to avoid the risk of bleeding. However, these results and those from a recent clinical trial have led to a rethink, as it is known that not all cases with low NIHSS have a good outcome [33].

In the analysis of factors influencing prognosis, as in other studies, the NIH stroke severity scale stands out [34]. Patients with low admission NIHSS show good prognosis, OR 3 to 7 times higher according to the outcome variable. Younger age is associated with a good prognosis at 1 year on the mRS and for mortality but not with the BI and the EQ-5D. Men have better prognosis when the outcome is measured with the BI and the EQ-5D. The EQ-5D results may be due to the fact that this difference is also found when analysing the general population [28]. Studies suggest that in general, the poorer HRQOL reported in older women is mainly due to a higher prevalence of disability and chronic diseases [35]. When quality of life was measured by the Visual Analogic Scale the results were near the same in the three measurements. This lack of sensitivity to change can be attributed to the limited validity of the thermometer to identify the preferences of stroke patients.

The quality of life at 1 year observed in our study is clearly higher (0.65) than that obtained in other Spanish studies conducted in 2000 and 2004 in which the average values were 0.49 and 0.50 respectively [16,36]. In the comparison of the characteristics of our sample with both studies we see that age and sex distributions were similar (mean age 72 years was and 55% of males). However the percentage of haemorrhages was lower in our study. As the sample sizes in both studies were small we need to be cautious to draw conclusions but it seems plausible that the changes found in terms of improved time to arrival at hospital, thrombolysis, rehabilitation and stroke units have had a direct bearing on the improvement in HRQOL. Despite these changes, the general population over age 65 still has an HRQOL 11% higher than the sample of patients with stroke. This difference, which can be interpreted as a clinically significant difference as it exceeds the range of 0.074 to 0.080 cited in the literature [37,38], reminds us that much more can still be done to minimise the functional impact of stroke. What is important is that many patients who suffer stroke have a very low HRQOL as a result of the loss of functional autonomy, and responding to this problem continues to be a challenge for public health [37]. The effect size of the intervention from discharge to the year is small according to the Cohen criteria [30]. However it is an important element to measure because it allows monitoring of the impact of tertiary stroke prevention. As the sample included some aphasic patients who were unable to communicate, the use of proxies was a limitation that we need to notice. The benefits of including proxy responses outweigh the loss of information that occurs when those patients are excluded, because the most impaired group is a key component of the prevalent stroke population [28].

The gender analyses can be interpreted as stroke units having reduced gender inequalities in care for stroke patients. We did not find the association between being female and higher mortality rates which has been reported by other studies [39] and there is no gender distinction in access to thrombolysis. The differences found in quality of life have also been described in the general population [30,35].

The age-standardised mortality rates for stroke have fallen worldwide over the past two decades [13]. However, despite this improvement in the epidemiological profile, we must not forget that stroke still represents one of the leading causes of death and disability. The absolute number of strokes, the prevalence of associated disability, the related deaths and the total global burden are large and have not come down [40,41]. Challenges remain, such as raising the percentage of patients receiving thrombolysis and controlling risk factors like hypertension or atrial fibrillation. The proportion of patients receiving thrombolytic treatment is very high (33%) but can still be improved. Identification of thrombolysis as a factor associated with good prognosis provides an incentive to maintain the goal of improving public awareness of stroke as a medical emergency. Additional options for this are the increased time window for thrombolysis and generalisation of endovascular treatment.

The three instruments applied on admission (NIHSS, Barthel and modified Rankin scales) showed good discriminating characteristics with measurements of good prognosis. In all cases the AUC exceeded 70% and tests of significance were statistically significant. Their use may make it possible to predict the functional outcome and thus help patients and families to prepare their response to the new social needs.

Quality indicators are currently collected in different European countries to monitor in the real world the quality of care received by stroke patients [42]. However, the huge variety in measuring the performance of stroke care hampers comparisons [43]. In the same line, our results describe how successful has been the implementation of strategies found effective in clinical trials when applied to Spanish hospitals.

Given that we did not include patients hospitalised in departments without stroke units, these results are not representative of all the Spanish stroke population. Thus, as a limitation we notice that these results are valid only for the percentage of stroke patients that are admitted into a stroke unit [44]. This figure has increased during the last years allowing the patients to receive a specific and proactive treatment. However, this study shows that decision-makers need to follow the recommendations from the Spanish Stroke Plan to benefit all the population and clearly promoting extension of the model to the majority of the more than 100,000 strokes that occur annually in Spain [45]. Expanding the coverage can be achieved by increasing the number of stroke units or through the use of telemedicine systems which allow the new techniques to be implemented even in rural areas where the population has poor access to tertiary hospitals such as those included in this study [46].

## Abbreviations

CONOCES: Costs and outcomes multicentre study of stroke patients admitted to a Stroke Unit in Spain
HRQOL: Health-related quality of life
NIHSS: National Institutes of Health Stroke Scale
ROC: Receiver operating characteristic
